# Classroom Immigrant Density Predicts Psychological Well-Being Among Adolescents With an Immigration Background: Findings From the 2017/18 Swedish Health Behaviour in School-Aged Children Study

**DOI:** 10.3389/ijph.2021.647380

**Published:** 2021-07-19

**Authors:** Maria Granvik Saminathen, Petra Löfstedt, Sara Brolin Låftman

**Affiliations:** ^1^ Centre for Health Equity Studies, Department of Public Health Sciences, Stockholm University, Stockholm, Sweden; ^2^ Unit for Mental Health, Children and Youth, Public Health Agency of Sweden, Stockholm, Sweden; ^3^ Department of Public Health and Community Medicine, Sahlgrenska Academy, University of Gothenburg, Gothenburg, Sweden

**Keywords:** immigrant density, psychological well-being, adolescence, Sweden, school

## Abstract

**Objectives:** Group-level characteristics in shared contexts such as schools may affect adolescent psychological health. This study examined if the immigrant density in the classroom was associated with the level of self-reported psychological complaints among students with an immigration background.

**Methods:** Cross-sectional data were derived from 2,619 lower secondary school students (aged 13–15) in the 2017/18 wave of the Swedish Health Behaviour in School-aged Children (HBSC) survey. Using multilevel analysis estimating two-level random intercept linear regression models, classroom immigrant density was considered as a potential predictor of immigrant students’ psychological complaints.

**Results:** Students with an immigration background reported significantly fewer psychological complaints, on average, than students without such a background, even when adjusting for other sociodemographic characteristics. A cross-level interaction indicated that both first- and second-generation immigrant students experienced fewer psychological complaints in immigrant-dense classes compared to when the proportion of immigrant students was lower.

**Conclusion:** Students with an immigration background fare better psychologically in classes with a higher proportion of immigrant students. Such compositional effects could be alleviated by strengthening all schools’ capacities to provide a more inclusive classroom climate.

## Introduction

The large-scale immigration experienced by countries like Sweden in the past decades has been accompanied by an increase in ethnic residential and school segregation [1]. Exposure to such structural inequalities, family socioeconomic deprivation [2] as well as challenges in negotiating identity and acculturation [3] has raised concerns about the social integration and psychological well-being of children and adolescents with an immigration background [4].

Depending on their reason for migration, country of origin, and education level, immigrant families encounter different obstacles to their mental health and well-being [5]. For instance, children of asylum seekers are considered to be at higher risk of poor psychological health, due to stressors related to displacement and resettlement [6]. Also, more generally, children and adolescents with an immigration background have been found to have higher rates of clinically diagnosed mental disorders [4]. Similar associations may apply to psychological problems more broadly. A meta-analysis of European studies showed that on average, immigrant children and youth present with higher levels of internalizing outcomes such as psychological distress and depressive symptoms than natives, especially at earlier stages of development [7].

Yet, other studies have found that children of immigrants in European countries like Sweden and elsewhere present with equal or better self-rated psychological well-being than their native peers [8]. This proposed advantage has partly been attributed to protective factors related to family conditions [8, 9]. Less attention has been placed on mechanisms related to group-level characteristics in shared contexts such as schools [10], which may promote student well-being by creating a positive psychosocial environment and providing social resources [11].

Children and adolescents’ well-being is shaped by factors in a variety of contexts. Ideological and institutional patterns are elements related to the macrosystem that can be decisive for people’s quality of life and future opportunities, for instance through social policies, while the family and the school are examples of spaces in the microsystem that play a role for young people’s outcomes [12]. As parental influence weakens during the transitional period of adolescence, peers tend to take on a more dominant role for the individual’s behavior and development [13]. Thus, peer relationships at school are essential for adolescents’ sense of belonging and level of well-being [14]. The social school environment may be particularly decisive for the well-being and future opportunities of students with an immigration background, as school presents an essential setting for sociocultural integration and interaction with majority group peers [15].

Increasing residential segregation and the “cream skimming” of children from high-SES families and the most motivated students into the most reputable schools through universal school choice has given rise to an uneven distribution of immigrant students into Swedish schools [1]. This has activated concerns about potentially harmful compositional effects. For instance, immigrant-dense schools have been suggested to negatively affect student academic achievement [16] and increase the risk of victimization [17], particularly for minority group students themselves [18]. Further, such environments may undermine successful acculturation and the development of a sense of belonging in the host society [19]. As a result, policy makers and educators alike tend to advocate for desegregation, with the aim of enhancing educational equity and student outcomes [20].

However, school characteristics that promote academic achievement may not be equally beneficial for adolescent psychological well-being [21, 22]. Attending schools and classes with a high proportion of native and/or more socioeconomically privileged peers could in fact be unfavorable for low-SES and immigrant students [23–25]. Accordingly, some studies have found that students with an immigration background report worse psychological well-being in schools with a majority of native students [26, 27]. One pathway explaining such an association may relate to social comparison mechanisms. Students from socially more disadvantaged backgrounds could feel academically inferior in high-SES schools that tend to have higher average academic achievement [28], in line with the “big-fish-little-pond” effect [29]. Experiencing a more competitive academic environment can also be expected to produce more stress related to school performance, which has in turn been associated with lower levels of psychological well-being [30].

Moreover, for adolescents with an immigration background, the theory of a protective ethnic density effect, established in the context of neighborhood influences on health [31], may apply in the school setting [32]. Ethnic minority populations have been shown to benefit from living in residential areas with a higher concentration of ethnic minority people [31], conceivably due to stronger social relationships [33] and less exposure to stigma related to their status as a minority [34]. In line with such theories, one potential mechanism accounting for the link between classroom immigrant density and the psychological well-being of immigrant students may be a stronger sense of immediate belonging and acceptance by peers [14].

During the developmental phase of adolescence, peers act as social mirrors, validating each other’s self-image [35]. Consequently, the affirmative experience of acceptance by peers in a central social setting such as the school can be decisive for the individual’s self-esteem [36], which is in turn linked with psychological well-being [37]. Students with an immigration background, particularly those with a more visible minority status, could find it more difficult to acquire group membership and a sense of belonging in classrooms with a high proportion of majority group students, as they may face more stigma related to their background in these contexts [38]. Such rejection by classmates in the school setting can be damaging for an adolescent’s self-image, and thus contribute to lower psychological well-being among immigrant students [26, 39]. By contrast, when surrounded by a high proportion of other students who are foreign-born or have foreign-born parents, students with an immigration background might have better chances of experiencing a stronger sense of belonging, in turn generating a higher level of well-being.

With such mechanisms in mind, the objective of this study was to test if (i) students with an immigration background generally reported less psychological complaints than students without an immigration background and if so (ii) to examine if this association was moderated by classroom immigrant density.

## Methods

### Study Sample

The study used data from the 2017/18 Swedish Health Behaviour in School-aged Children (HBSC) study, a cross-sectional survey among 11-, 13-, and 15-year-olds that is part of an international World Health Organization collaborative study. The survey was conducted in grades 5, 7, and 9, with schools being randomly selected from all public and independent schools in Sweden for each grade, where after one class per school was randomly sampled. The questionnaires were distributed anonymously to all students present in class on the day of the survey.

As this study focused on lower secondary school, only students in grades 7 and 9 were included (*n* = 3,095 distributed across 145 school classes). The final study sample was restricted to students with complete information for the relevant variables (*n* = 2,619 distributed over 145 school classes).

In the data, 239 students attended schools where no participating students reported having an immigration background. As a sensitivity check (not presented in table), we ran all analyses excluding these students. The results from these additional analyses remained almost identical to those presented.

### Measures

*Psychological complaints* were operationalized as an index of four items. Participants indicated how frequently they experienced each of the following symptoms: feeling low, feeling nervous, irritability or bad temper, and difficulties in getting to sleep. Four response options were provided for each question: “about every day”, “more than once a week”, “about every week”, “about every month”, and “rarely or never.” A summary scale with a range of 4–20 was developed, with a higher score indicating a higher frequency of psychological complaints. A Canadian study that examined the external construct validity of the HBSC psychological health self-report question series found that the four items included in the measure of psychological complaints used in this study presented as one single factor [40].

*Immigration background* differentiated between students who reported being born abroad and/or having two parents who were born abroad, and students without an immigration background. A more refined version of this variable further differentiated between first- (students born abroad) and second-generation immigrant students (students born in Sweden with two foreign-born parents).

*Classroom immigrant density* was measured as the share of students with immigration background in the class in per cent.

The analyses were adjusted by several control variables. The *Family Affluence Scale (FAS)* is a measure reflecting family expenditure and consumption used to identify the socioeconomic status of adolescents. This study used version III of FAS [41], an index consisting of six items: family car ownership; having one’s own bedroom; number of computers (including laptops and tablets) in the household; number of bathrooms in the household; ownership of dishwasher; number of times travelled abroad on holiday in last year. Responses to the items were summed up and calculated as an index ranging from 0–13.

*Family structure* differentiated between students who explicitly reported living with both parents (coded 1) and those who alternated between two parents, lived with only one parent, or in any other circumstances (coded 0).

We further controlled for *school grad*e (year 7 or 9) and student *gender* (boy or girl).

### Statistical Analysis

Analyses were performed using Stata version 14.2 (StataCorp LLC, College Station, TX, United States). The hierarchical nature of the data with students nested in school classes warranted a multilevel approach [42] A series of two-level random intercept linear regression models were run using the “mixed” command, examining the role of immigration background and classroom immigrant density for psychological complaints. Model 1 estimated the level of psychological complaints of students with an immigration background compared to those without such a background, controlling for school grade, student gender, family structure and socioeconomic status. Model 2 further adjusted for classroom immigrant density. Model 3 added a cross-level interaction between student immigration background and classroom immigrant density. Intra Class Correlation coefficients (ICC), indicating the amount of variation that can be attributed to the higher level, were reported for all models.

### Ethical Considerations

The Swedish HBSC data does not contain any personal identification and the questionnaire is completed anonymously and voluntarily by the participants. Informed consent was obtained from the students who participated. Parents/guardians were informed about the study by the schools and parent who did not want their children to participate were asked to inform the school. The collection of data with the Swedish HBSC survey was not classified as personal data and thus this study was deemed exempt from human subjects review by the Regional Ethical Review Board in Stockholm, Sweden.

## Results

As shown in Table 1, immigrant students reported significantly fewer psychological complaints, on average (10.21), than students without an immigration background (11.04). Further, students with an immigration background had a significantly lower family socioeconomic status (14.61) than students without an immigration background (15.69), on average. The average immigrant density of a student’s respective school class was significantly higher for immigration background students (46.70) than for students without an immigration background (17.49). While 38.6% of immigrant students were part of classes with an immigrant density of at least 50%, the corresponding share for students without an immigration background was 4.0% (data not presented). Finally, a significantly larger proportion of students without an immigration background reported living with both parents (69.8%) than among students with an immigration background (65.3%).

**TABLE 1:** Descriptive statistics of the variables for the total study sample and by immigration background (based on 2,619 students distributed over 145 school classes). Health Behavior in School-aged Children study, Sweden, 2017/18.

	All (*n* = 2,619)			No immigration background (*n* = 2,039)			Immigration background (*n* = 580)			Sig. diff.^a^
	Mean	*SD*	Range	Mean	*SD*	Range	Mean	*SD*	Range	t-test
Psychological complaints										
Unstandardized	10.85	4.02	4–20	11.04	3.96	4–20	10.21	4.18	4–20	*p* < 0.001
Standardized^b^	0.007	1.00	−1.70–2.27	0.05	0.98	1.70–2.27	-0.15	1.04	1.70–2.27	—
Family affluence scale	15.44	1.96	6–19	15.69	1.72	8–19	14.61	2.45	6–19	*p* < 0.001
Classroom immigrant density	23.96	22.76	0–100	17.49	16.14	0–91.67	46.70	27.58	3.70–100	*p* < 0.001

	*N*	%		*N*	%		*N*	%		χ^2^
Grade										
7	1,174	44.8	—	915	44.9	—	259	44.7	—	*p* = 0.925
9	1,445	55.2	—	1,124	55.1	—	321	55.3	—	
Gender										
Boys	1,256	48.0	—	988	48.5	—	268	46.2	—	*p* = 0.339
Girls	1,363	52.0	—	1,051	51.5	—	312	53.8	—	
Family structure (live with both parents)										
Yes	1,803	68.8	—	1,424	69.8	—	379	65.3	—	—
No	816	31.2	—	615	30.2	—	201	34.7	—	*p* = 0.039

a Two-sample t-tests for continuous variables and Pearson’s *χ* ^2^ tests for categorical variables.

b The analyses in this study are based on the unstandardized version of psychological complaints.

Table 2 presents results from random intercept models with psychological complaints as the dependent variable.

**TABLE 2:** Results from two-level random intercept linear regression models (b coefficients). Student-reported psychological complaints according to immigration background (based on 2,619 students distributed across 145 school classes). Health Behavior in School-aged Children study, Sweden, 2017/18.

	Model 1	Model 2	Model 3
*Student-level*			
No immigration background	0	0	0
Immigration background	−0.941^***^	−0.907^***^	−0.152 (n.s.)
*Classroom-level*			
Classroom immigrant density	—	−0.002 (n.s.)	0.009 (n.s.)
*Cross-level interaction*			
Immigration background^*^Classroom immigrant density	—	—	−0.023^**^
*ICC*	0.038	0.038	0.035

* *p* ≤ 0.05.

** *p* ≤ 0.01.

*** *p* ≤ 0.001.

Model 1: Adjusted for school year, gender, family structure, and socioeconomic status. Model 2: Model 1 + classroom immigrant density. Model 3: Model 2 + cross-level interaction between immigration background and classroom immigrant density.

The intraclass correlation coefficient (ICC) for Model 1 shows that only 3.8% of the variance in adolescents’ psychological complaints is accounted for by school-level, rather than student-level, differences (ICC = 0.038), indicating that there is generally a rather low similarity between students from the same school with regards to psychological complaints.

As a first step, differences in levels of psychological complaints between immigrant and non-immigrant students were reviewed (Model 1). Even when adjusting for school grade, gender, family structure and socioeconomic status, students with an immigration background reported significantly fewer psychological complaints (b = −0.941, *p* ≤ 0.001, 95% CI −1.31, −0.57) on average, than students without an immigration background. This advantage was lowered to −0.907 (*p* ≤ 0.001, 95% CI −1.32, −0.50) when further adjusting for classroom proportion of immigrant students, although this class-level measure did not show a statistically significant association with psychological complaints (b = −0.002, *p* = 0.692, 95% CI −0.01, 0.01) (Model 2). Next, a cross-level interaction between individual immigration background and classroom immigrant density was added (Model 3). This interaction term was negative and statistically significant (b = −0.023, *p* = 0.006, 95% CI −0.04, −0.007), conveying that the association between individual student immigration background and the level of psychological complaints varied based on the proportion of immigrant students in the classroom. More specifically, this result demonstrates that students with an immigration background experienced fewer psychological complaints when in classes with a higher proportion of immigrant students compared to being in classes with a low immigrant density.

Table 3 presents the same models as in Table 2 while using a more refined version of the independent variable, distinguishing between first- and second-generation immigrants. Both first- (b = −1.08, *p* ≤ 0.001, 95% CI −1.57, −0.60) and second-generation immigrants (b = −0.83, *p* ≤ 0.001, 95% CI −1.32, −0.34) reported lower levels of psychological complaints, on average, than students without any immigration background (Model 1). These associations were slightly reduced for both first- (b = −1.05, *p* ≤ 0.001, 95% CI −1.55, −0.54) and second-generation (b = −0.78, *p* ≤ 0.001, 95% CI −1.31, −0.25) immigrant groups in Model 2. The cross-level interaction was also statistically significant for both immigrant background groups, suggesting that the finding regarding the role of class immigrant density applies to both first- (b = −0.021, *p* = 0.037, 95% CI −0.04, −0.001) and second-generation immigrants (b = −0.026, *p* = 0.008, 95% CI −0.05, −0.007).

**TABLE 3:** Results from two-level random intercept linear regression models (b coefficients). Student-reported psychological complaints according to first- and second-generation immigrant status (based on 2,612^a^ students distributed across 145 school classes). Health Behavior in School-aged Children study, Sweden, 2017/18.

	Model 1	Model 2	Model 3
*Student-level*			
No immigration background	0	0	0
First-generation immigrant	-1.08^***^	-1.05^***^	-0.41 (n.s.)
Second-generation immigrant	-0.83^**^	-0.78^**^	0.19 (n.s.)
*Classroom-level*			
Classroom immigrant density	—	-0.002 (n.s.)	0.009 (n.s.)
*Cross-level interaction*			
Immigration background^*^Classroom immigrant density	—	—	—
First-generation immigrant	—	—	-0.021^*^
Second-generation immigrant	—	—	-0.026^**^
*ICC*	0.035	0.035	0.034

a 7 participants had missing information on the variable *country of birth*.

* *p* ≤ 0.05.

** *p* ≤ 0.01.

*** *p* ≤ 0.001.

Model 1: Adjusted for school year, gender, family structure, and socioeconomic status. Model 2: Model 1 + classroom immigrant density. Model 3: Model 2 + cross-level interaction between immigration background and classroom immigrant density.

## Discussion

The results of the current study corroborate previous research that has suggested an advantage in self-reported psychological well-being among children of immigrants in Sweden [8]. With respect to immigrant density, the study found that adolescents with an immigration background experienced fewer psychological complaints when in school classes with a higher proportion of other immigrant students than in classes with a majority of non-immigrant students. Although these findings are not able to account for the levels of psychological well-being of specific groups of immigrants, such as children of refugees [6], these associations were shown to be of relevance for both first- and second-generation immigrants.

The findings regarding the association between classroom immigrant density and the level of student psychological complaints are in line with a previous Swedish study by Hjern et al. [27] which showed that specifically migrant students born in Africa or Asia reported more positive psychological well-being in classes with a high density of students with a migrant origin. Similar mechanisms that account for group density effects in communities at large [31] may thus be relevant for adolescents in schools.

The examination of immigrant density in schools is particularly relevant considering the extensive immigration to countries like Sweden in the past decade [43], which has exacerbated the spatial segregation of different sociodemographic groups and the corresponding uneven distribution of children into schools [1]. As reflected in the most recent Swedish HBSC data, a large proportion of immigrant students are indeed likely to attend schools with a high immigrant density. Notwithstanding, some immigrant families choose to place their children in schools with a majority of native students that tend to be perceived as more prestigious, at times in search of a more advantageous “Swedish” learning environment [44].

Yet, having an immigration background may render students more vulnerable to social exclusion in such school settings, as previous studies have indicated. For instance, a Swedish study by Plenty and Jonsson [38] found that students with an immigration background had higher peer rejection rates than native students, particularly in classes with a low immigrant density. Likewise, another study based on Swedish data showed that students born in Africa and Asia attending classes with few other immigrants were at high risk of being bullied [27]. Such negative judgements by peers, signaling low acceptance, can in turn be decisive for an individual’s self-esteem and psychological well-being. Thus, while attending school with a high proportion of majority group students could potentially be advantageous for immigrant students in terms of social integration, the acquisition of the local language and improved academic achievement [45], they may struggle more to find a sense of acceptance and belonging than in classes with a higher proportion of fellow immigrants. As a consequence of the deeply rooted need to be accepted by peers [36], the existing segregated school landscape in Sweden may thus discourage adolescents with an immigration background to apply to schools with a lower immigrant density, further reinforcing school segregation and undermining the integration into the majority society.

Aside from processes related to peer acceptance, there are other micro-level mechanisms that may account for the proposed association between immigrant density and psychological complaints among immigration background students. For instance, some students with an immigration background could find themselves at an academic disadvantage when compared to native classmates, particularly those students who have recently immigrated and/or have a less socioeconomically privileged family background. Further, students with a more socially disadvantaged background may not be able to fully benefit academically from a relatively more effective learning context when attending more socially privileged schools, as these schools may not always provide appropriate academic and psychosocial support to such student groups. Thus, social comparison mechanisms could have negative implications for the academic self-concept of immigrant students in schools with a high proportion of native Swedish students [28], with potentially adverse short- and long-term effects on well-being.

Ultimately, the findings of this study suggest that the transfer of individual immigrant students to majority-dense schools, for instance through the universal school voucher system established in Sweden [1, 44], may not necessarily benefit the immediate psychological well-being of this group of adolescents, despite potential academic benefits [21]. However, even if adolescents with an immigration background may present with fewer psychological complaints when they attend schools and classes with many other immigrant students, this may not be an indication of equally positive outcomes in other important areas such as educational achievements and employment prospects. Here, factors at the macro-level such as housing segregation must be acknowledged as challenges that undermine the successful integration of immigrant families and may end up standing in the way of adolescents’ future opportunities.

### Limitations

The use of HBSC data provides a valuable opportunity to map patterns of adolescent health and well-being. However, due to the cross-sectional nature of the HBSC surveys, we cannot draw any causal conclusions based on our findings. Further, some shortcomings of this study have limited the scope of our analyses. Firstly, as mentioned in the Introduction, research findings on the relative psychological well-being of children and adolescents with an immigration background are divided, and several studies have determined that immigrant children generally fare worse than children of natives [7]. For instance, children of certain groups of immigrants, such as asylum-seekers, are known to be at high risk for mental and psychosocial problems [46]. However, the Swedish HBSC survey does not provide information on country of origin, time since immigration, or the reasons for migration, so that it was not possible to uncover potential groups of immigrants that could be driving the established relationship [27]. Additional analyses (not presented in table) that distinguished between immigrants of European and non-European origin showed that classroom immigrant density was statistically significantly associated with psychological complaints for students of non-European origin, but not for those with a European background However, since only 2.5% (*n* = 66) of our sample reported having a background in another European country, we opted to merge this group with students of non-European origin. Furthermore, records of student achievement or ability would have been useful as control or potential mediation variables. Finally, the outcome variable psychological complaints was operationalized as a summary scale, thus not accounting for the categorical nature of the individual items. Further, it should be stressed that the outcome variable captures self-reported symptoms that do not reflect clinical diagnoses, implying that the identified advantage in psychological well-being among adolescents with an immigration background cannot be generalized to the prevalence of more serious psychiatric disorders that may require treatment [47].

### Conclusion

This study investigated the potential role of classroom immigrant density for immigrant students’ psychological complaints. The results show that adolescents in Sweden who were foreign-born or had two foreign-born parents reported fewer psychological complaints when in school classes with a higher immigrant density than in classes with a low proportion of immigrant students. This association were shown to apply to both first- and second-generation immigrant students.

The findings suggest that efforts to cultivate a more inclusive social classroom climate may be able to mitigate compositional effects and thus promote the psychological well-being of adolescents with an immigration background, even in a segregated school landscape. Accordingly, policy makers and schools should become more aware of class compositional effects that may affect the general psychological well-being of students with an immigration background. While the distribution of students into schools is determined by factors at the macro-level and thus largely beyond the control of schools themselves, an increased awareness of the importance of feeling accepted in the classroom, particularly for minorities, is vital for the successful integration and academic attainment of all students. Thus, all schools should be provided with resources and tools to develop an inclusive academic and social climate where students are less likely to be excluded or stigmatized based on their ethnic or immigration background [15, 20] Yet, structural responses may be required to break current segregation patterns and to improve future opportunities for children of immigrants.

To reinforce such objectives, future studies should explore evidence regarding potential mechanisms that could account for the association between the sociodemographic classroom composition and student well-being. For instance, certain school and classroom characteristics could have a buffering effect that could improve the sense of belonging of immigrant students, even in majority-dense classrooms. It may also be valuable to observe the development of the significance of immigrant density in schools over time, following the recent inflow of migrants and refugees in the past few years. Finally, there is a need for more detailed studies of different groups of immigrants, for example based on country or region of origin, in order to ensure that students who may be particularly vulnerable to exclusion and victimization at school can be acknowledged and effectively supported.

## Data Availability

The data analyzed in this study is subject to the following licenses/restrictions: Data can be requested from the Public Health Agency of Sweden. Requests to access these datasets should be directed to petra.lofstedt@folkhalsomyndigheten.se.
